# Mental health care use of autistic adults: Identifying longitudinal patterns using sequence analysis

**DOI:** 10.1177/13623613241304513

**Published:** 2024-12-18

**Authors:** Iris Selten, Tim Ziermans, Iris Rapoport, Kim Jonkman, Hilde M Geurts

**Affiliations:** 1 University of Amsterdam, The Netherlands; 2 Vrije Universiteit Amsterdam, The Netherlands; 3 Leo Kannerhuis (Youz/Parnassia Groep), The Netherlands

**Keywords:** autism spectrum disorders, health services, interventions, pharmacologic, psychiatric comorbidity, psychosocial/behavioral

## Abstract

To improve mental health care for autistic adults, a better understanding of their real-world intervention use is required. The aim of this study is to investigate individual differences in the intervention trajectories of autistic adults, by examining longitudinal patterns of therapy, counseling, and medication use. Self-reported intervention use of 445 autistic adults (18–87 years) across at least five consecutive measurements (2015–2021) was collected to identify clusters using sequence analysis and hierarchical clustering. Sensitivity analyses and cross-method validation (group-based multivariate trajectory modeling) were used to verify the obtained clusters. Four out of five obtained clusters had sufficient internal consistency and were replicated. These four clusters had distinct intervention trajectories (least intervention, mostly counseling, mostly medication, mixed use) and differed on several demographic variables. Most notably, the “mixed cluster” contained relatively more females and individuals with self-reported co-occurring psychiatric conditions. Many autistic adults used interventions, although they varied in type and duration of intervention use. This reflects the clinical complexity of tailoring intervention approaches and highlights that accessible and multidisciplinary care is essential to adequately support autistic adults. An important next step is to investigate whether available interventions sufficiently support autistic females and those with co-occurring psychiatric conditions.

**Lay abstract:**

Many autistic adults experience co-occurring mental health problems, which have a negative effect on their well-being and result in increased use of mental health services. To improve mental healthcare for autistic adults, a better understanding is needed of what type of support they use in real life. Clinical guidelines recommend three kinds of mental health interventions: therapy, counseling, and medication. We investigated the use of these types of interventions in a sample of 445 autistic adults (aged 18–87 years) across a 5-to-7-year period. We found evidence for four different patterns of intervention use, or so-called subgroups: (1) *least intervention use*, (2) *mostly counseling*, (3) *mostly medication*, and (4) *mixed intervention use*. The group with mixed intervention use consisted of relatively more females and individuals with co-occurring psychiatric conditions, especially compared to the subgroup with the least intervention use. It appeared that many, but not all, autistic individuals used mental health services for an extended period. However, there was considerable variability in the type, combination, and duration of intervention use. This means that determining the optimal support for autistic adults is often a complex task, which requires collaboration of clinical experts and autistic individuals themselves, to make informed decisions.

Autism spectrum disorder (hereafter referred to as Autism) is considered a lifelong condition that can also be identified in adulthood (Baxter et al., 2015; Howlin, 2021; Lai & Baron-Cohen, 2015). More than half of the autistic adults^1^ experience co-occurring mental health problems (Lai et al., 2019), which significantly impact their well-being (Oakley et al., 2021; van Heijst & Geurts, 2015) and result in high rates of mental health service use (Weiss et al., 2018; Zerbo et al., 2019). Accessible and adequate mental health interventions are therefore crucial to both improve individual outcomes, and reduce societal mental health care costs (Masi et al., 2017). A comprehensive description of the real-world mental health care utilization of autistic adults can provide leads to optimize both policy and clinical practice (Shoaib et al., 2022). The present study, therefore, aims to shed light on longitudinal patterns of real-world mental health care use in a Dutch community sample of autistic adults.

In the Netherlands,^2^ in line with international guidelines, mental health care for autistic adults primarily encompasses three kinds of interventions: therapy, counseling, and medication (Akwa GGZ, 2017; Lord et al., 2022; Maddox et al., 2021; National Institute for Health and Care Excellence [NICE], 2012; NVvP & NIP, 2013). Therapy involves structured interventions offered by a licensed clinician (Benevides et al., 2020; White et al., 2018), whereas counseling refers to coaching in daily-life or practical situations, such as providing vocational support or assistance with financial administration (Nicholas et al., 2015). Medication is implemented to target co-occurring psychiatric problems in autistic individuals, rather than core autism-associated symptoms (Jobski et al., 2017). Varying rates of such intervention use have been reported, ranging from 30% to 50% of autistic individuals using therapy, between 25% and 80% using counseling, and between 45% and 70% using medication (Buck et al., 2014; Cvejic et al., 2018; Dudley et al., 2019; Esbensen et al., 2009; Feroe et al., 2021; Höfer et al., 2022; Houghton et al., 2017, 2018; Ishler et al., 2022; Płatos & Pisula, 2019; Schott et al., 2021; Shoaib et al., 2022; Turcotte et al., 2016; Vogan et al., 2017).

However, caution is warranted in interpreting these findings, as many studies focused only on relatively young adults (mean age ~25 years; Ishler et al., 2022; Schott et al., 2021; Turcotte et al., 2016), did not differentiate between children and adults (Esbensen et al., 2009; Houghton et al., 2017, 2018; Płatos & Pisula, 2019; Shoaib et al., 2022) or between autistic individuals with and without intellectual disability (Buck et al., 2014; Gotham et al., 2015; Höfer et al., 2022). In addition, a substantial number of reports used health-claims data to describe intervention use (Feroe et al., 2021; Houghton et al., 2017, 2018; Schott et al., 2021; Shoaib et al., 2022), which biases the results toward autistic adults seeking help. Furthermore, despite reports that autistic individuals used therapy concurrently with either counseling or medication (Cvejic et al., 2018; Feroe et al., 2021; Houghton et al., 2017, 2018; Shoaib et al., 2022; Vogan et al., 2017), use of all three types of intervention has not been investigated in a single study. Finally, evidence exists that autistic adults used interventions for a prolonged period (Cvejic et al., 2018; Esbensen et al., 2009; Feroe et al., 2021; Shoaib et al., 2022). However, longitudinal studies are limited, focused mostly on medication use and covered a short period (i.e. 3–4 years). This underscores the need of further comprehensive understanding of the real-world intervention use of autistic adults, particularly focusing on the longitudinal use of different kinds of intervention.

Autism is characterized by heterogeneity, meaning that autistic individuals vary in socio-demographic background, living situation, cognitive and adaptive functioning, and their presentation of autistic features and co-occurring mental health problems (Masi et al., 2017). As such, this requires the intervention approach to vary between autistic individuals, posing a challenge to tailor intervention in clinical practice. Studying real-life intervention use allows for a further illustration of the existing variation in intervention use among autistic adults and a better understanding of which factors contribute to this variation.

The present study aims to complement previous studies into the intervention use of autistic adults. To this end, we will examine intervention trajectories in a community sample of autistic adults without intellectual disability. Intervention trajectories are defined as an individual’s use of therapy, counseling, and medication over a period of 5–7 years. To shed light on variation in intervention use, we aim to detect the most dominant intervention trajectories by identifying subgroups of autistic adults in our sample, based on their intervention trajectory. To support the interpretability of the results, we will describe and label the trajectories of each subgroup. For instance, we might detect a subgroup with an intervention trajectory that is characterized by re-using intervention after a period without intervention, and we may label this a “re-use trajectory.” Finally, we will describe the distinct characteristics of each subgroup.

## Method

### Participants and procedure

This study used data from the Netherlands Autism Registry (NAR), a database containing information of autistic individuals in the Netherlands. Recruitment advertisements in local and national media, both offline and online, were used to recruit autistic individuals for participation in the NAR. Individuals can register on the NAR website (https://www.nederlandsautismeregister.nl/english/), after which they receive yearly invitations to fill out the NAR questionnaires. Every year a participant can decline (further) participation.

The NAR has been approved by the ethical review board of the Vrije Universiteit Amsterdam (VCWE 2020-041R1). All participants provided written informed consent. Participants were requested to indicate where (i.e. which institute), when (i.e. which year), and by whom (i.e. which type of professional) they received their autism classification. This enabled us to verify that classifications were established by a licensed professional. The NAR questionnaires are constructed by autistic and non-autistic members of the research team, which increases the likelihood that both autistic and non-autistic individuals have a similar interpretation of the questions and answer options.

In NAR questionnaires, participants are requested to report retrospectively. For this study, we used data that were collected between 2016 and 2022, implying that we received information on intervention use in the years 2015–2021. Out of a “total sample” of autistic adults (*n* = 2166; 18–78 years) without intellectual disability, we identified our “study sample” of 445 participants that met the inclusion criteria for further analyses (Step 1 of the analyses section; Table 1).

**Table 1.** Characteristics of the total sample and study sample and comparisons between these samples.

Variable	Total sample	Study sample	Statistics		
	*N* = 2166	*N* = 445	*F/*χ^2^	*p*	*V/*ɳ^2^
Biological sex			1.70	0.193	0.03
Female	1274 (59%)	251 (56%)			
Male	868 (40%)	192 (43%)			
Other	23 (1%)	2 (0.5%)			
Not available	1 (<1%)	-			
Age in years			53.3	<0.001	0.02
Mean (SD)	44.7 (13.8)	49.0 (13.5)			
Range	18.0-87.0	18.0-85.0			
Age of diagnosis in years			0.64	0.423	0.00
Mean (SD)	36.3 (15.0)	36.8 (15.3)			
Range	0.67-75.5	2.17-75.5			
Not available	124 (6%)	409 (8%)			
Time since diagnosis in years			168.2	<0.001	0.08
Mean (SD)	8.32 (6.44)	12.0 (5.74)			
Range	0.00-60.0	1.17-34.7			
Not available	22 (1%)	409 (8%)			
Autism Quotient (AQ; 28-items)			2.62	0.133	0.00
Mean (SD)	83.0 (11.1)	83.8 (10.7)			
Range	44-110	50-109			
Not available	90 (4%)	12 (3%)			
*N* > cut-off 65	2028 (93%)	423 (95%)			
Ethnicity			0.08	0.776	0.01
Dutch	2040 (94%)	422 (95%)			
Other	56 (3%)	12 (3%)			
Not available	69 (3%)	8 (2%)			
Educational level^a^			3.27	0.195	0.05
Low	186 (9%)	30 (7%)			
Middle	542 (25%)	116 (26%)			
High	884 (41%)	195 (44%)			
Not available	554 (26%)	104 (23%)			
Co-occurring psychiatric condition^b^			10.8	0.001	0.07
None	1024 (47%)	242 (54%)			
1 or more	1035 (48%)	183 (41%)			
Not available	107 (5%)	20 (4%)			

All information is based on self-report, including co-occurring psychiatric conditions.

a Educational level was classified in accordance with Statistics Netherlands (CBS).

b Individuals indicated to have a co-occurring psychiatric diagnosis at the time of their NAR participation.

### Measures

#### Intervention use

Participants’ intervention use was measured with three binary questions (i.e. “Did you use therapy/counseling/medication related to autism in the past year or since you filled out the last questionnaire?”). The difference between therapy and counseling was made explicit in the questions.^3^ Furthermore, participants received a multiple-choice question to indicate the types of therapy, counseling, and medication they used. For medication, this list only included psychopharmacological drugs. This way, we controlled that participants did not report on health care use associated with physical complaints. For therapy only, the duration of intervention use in months was requested.

#### Demographic information

Participants reported on demographic characteristics, including biological sex, educational level, employment and relationship status, and co-occurring psychiatric conditions.

### Analyses

Analyses were done in RStudio version 4.2.2 (RStudio Team, 2020) using packages Tidyverse (Wickham et al., 2019), TraMineR (Gabadinho et al., 2011), and GBMT (Magrini, 2022). Our analyses were preregistered at AsPredicted.org (#133884 and #133889). If we deviated from our preregistered analyses plan, it is explicitly reported. We used state sequence analysis (SA), as our primary method to identify clusters of autistic adults based on their intervention trajectory (Liao et al., 2022; Nguefack et al., 2020). Using SA terms, intervention trajectories are defined as *sequences of states*, with each state referring to the type of intervention use in each year. The analytical strategy consisted of six steps, which are further explained below.

#### Step 1. Defining the study sample

SA provides the most meaningful results when the number of missing states and inter-individual differences in state-sequence length remain limited (Liao et al., 2022). First, we aimed to fill missing data points for each individual. Given that some of the missing data may be missing not at random and missing data occurred in our outcome variable, we did not deem multiple imputations a suitable approach to handle missing data. Instead, we manually filled missing data points in intermittent years, based on available data in surrounding years (Figure S1; Table S1 for explanation). In addition, we decided that the maximum inter-individual difference in sequence lengths could not be more than two states. We preregistered to use NAR data gathered from 2015 to 2022 (i.e. reported on 2014–2021), resulting in a longest possible sequence length of eight states. As information on counseling was, unfortunately, not requested in 2015, we used data gathered from 2016 to 2022 (i.e. reported on 2015–2021). As such, the longest possible sequence consisted of seven states. Thus, only those participants in the total sample with data on intervention use on *at least five consecutive years* were included in the study sample.

#### Step 2. Defining states and constructing state sequences

For each individual, their state sequence (*intervention trajectory*) consisted of consecutive states that were measured each year (i.e. 2015–2021; Table 2). As all participants had data on at least five consecutive measurement waves, states at the beginning (years 2015–2016) and/or end of a sequence (years 2020–2021) could not be defined for all participants. It was decided to left-align all individual sequences, as we were interested in the *order* of different states, rather than their *timing*. This implies that all sequences started at Time point 1 (T1), regardless of the chronological year that T1 was measured. Likewise, all sequences ended at T5, T6, or T7, whereas these time points did not correspond to the same chronological year. For instance, an individual who participated in the NAR in 2015 until 2021 has their T1 in 2015 and T7 in 2021, whereas an individual participating from 2016 to 2020 has their T1 in 2016 and T5 in 2020.

**Table 2.** Different states and their abbreviations.

State		Abbreviation
1	No intervention use	No
2	Using therapy	T
3	Using counseling	C
4	Using medication	M
5	Using therapy and counseling	T&C
6	Using therapy and medication	T&M
7	Using counseling and medication	C&M
8	Using therapy, counseling, and medication	T&C&M
*(9)*	*(Missing)*	*(M)*

#### Step 3. Measuring similarity between sequences

A dissimilarity matrix was created to examine (dis)similarities between the individual state sequences. We used a distance metric that is based on Longest Common Subsequences (LCS; Elzinga, 2007), as this measure gives priority to the similarity of the order of events in the data, above chronological similarity, thereby fitting the main interest of this study. Finally, we used a normalization procedure of the dissimilarities that accounts for the minor differences in sequence length (Gabadinho et al., 2011).

#### Step 4. Clustering of sequences

Agglomerative hierarchical clustering with Ward’s criterion was employed to identify clusters of individuals based on the dissimilarity matrix. In other words, individuals were clustered based on similarities in their state sequence (i.e. intervention trajectory). To decide on the final number of clusters in the data, we discarded cluster solutions if any one of the clusters includes *n* < 10% of the total sample. We evaluated the measure of weighted average silhouette width (ASWw), which reflects the overall consistency of the clusters. It fluctuates between −1 and 1, with a value close to 1 indicating larger inter-cluster distances and stronger intra-cluster homogeneity. We follow Kaufman and Rousseeuw (1990; as summarized in Studer, 2013) for interpretation of the ASWw values. Subsequently, we bootstrapped the data and computed the Jaccard similarities (JC) as a measure of consistency of clusters. For interpretation of the JC values, we followed Hennig (2008).

#### Step 5. Sensitivity analyses

First, we examined the possible influence the COVID-19 pandemic, as this may have influenced the access to mental health care for autistic adults (Scheeren et al., 2023). To this end, we re-conducted the original SA analysis without the data obtained in 2020 and 2021. If results of this analysis differ from the original analysis, this could indicate that the COVID-19 pandemic induced a different pattern of intervention use.

Second, we examined the effect of missing data that are missing *not* at random, as this type of missing data may particularly negatively impact the validity and generalizability of the results of the SA analyses (Liao et al., 2022). Given that individuals can decide each year if they take part in NAR, the individuals with missing data at recent years might differ from those who continue to participate in NAR. We conducted a second sensitivity analysis, in which we added a specific state, to code missing states as “missing” (Table 2, State 9). If results of this analysis differ from the original analysis, this could indicate that our results are biased by attrition.

#### Step 6. Cross-method validation

Cross-method validation is recommended to confirm the results of an initial clustering analysis (Agelink van Rentergem et al., 2021). We employed group-based multivariate trajectory (GBMT) modeling in the same dataset, using the same outcomes of intervention use (Magrini, 2022; Nagin et al., 2018). GBMT identifies groups of individuals following similar trajectories over time for multiple outcomes, and it is suited for analysis of categorical longitudinal data. Here, we slightly deviated from our preregistration. That is, we preregistered that we would conduct all steps of both SA and GBMT analyses. However, given that GBMT was used as cross-method validation for the SA results, the first steps of the GBMT model became redundant as we only needed to test the range of GBMT models that corresponded to the models that were tested in SA. We use Bayesian information criterion (BIC) and Akaike information criterion (AIC) to compare these different models. We specified four criteria that needed to be met to consider a GBMT-Subgroup to be similar to an SA-Cluster: (1) representing a similar intervention trajectory; (2) having the same outcome on at least half of the years for each kind of intervention; (3) consisting of at least 75% of the same participants; and (4) sharing a profile of demographic variables.

#### Step 7. Cluster descriptives

We compared the different clusters on demographic variables using chi-square tests or analysis of variance (ANOVA) and applied Bonferroni correction to correct for multiple comparisons.

### Community involvement

The NAR team consists of both non-autistic and autistic individuals, and consults autistic adults for input on the NAR questionnaires. In addition, an in-person meeting was organized to share our results with three autistic adults, who received a financial reward, and two clinical psychologists affiliated with a mental health care clinic specialized in care for autistic people. Together we discussed their experiences with interventions, which contributed to the interpretation of our results and thinking of future directions for this research.

## Results

### Sample description

The total sample consisted of relatively many females (Loomes et al., 2017) and few individuals with a low educational level (8.6%), as approximately 26% of the Dutch population is estimated to belong to this category (CBS, 2021). As aforementioned, only those individuals within the total sample that had data on *at least 5 consecutive measurement points* were included in our further analyses (study sample, *N* = 445). Statistical comparisons showed that individuals in the study sample were on average older, had a longer time since they received their autism diagnosis, and less frequently had a co-occurring psychiatric condition (Table 1).

**Figure 1.**
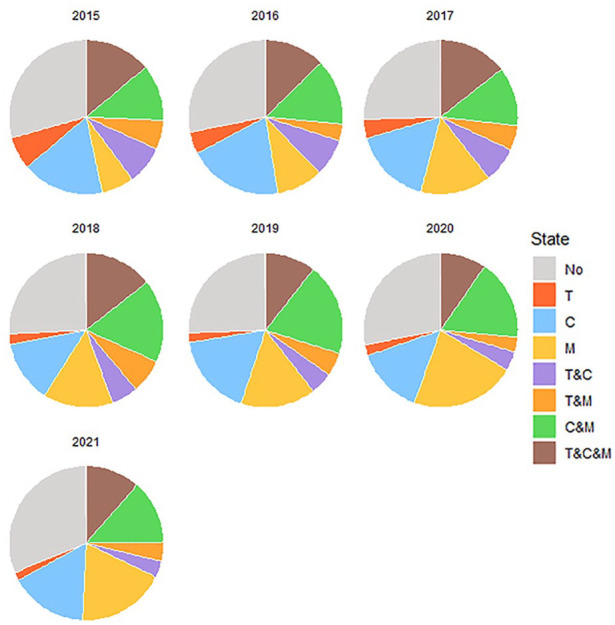
Proportion of individuals using each kind of intervention for each year for the study sample (*N* = 445). T: therapy; C: counseling; M: medication.

The study sample consisted of individuals with at least one missing data point (*n* = 244) and with complete data on all seven data points (*n* = 201). Participants with complete data had their autism diagnosis for a shorter time (*M* = 10.7 years) than participants with incomplete data (*M* = 13.0 years). Furthermore, the proportion of male participants was larger among the participants with complete data. Participants with missing data in the final years (2020/2021) were compared to all other participants, as these data points were considered missing not at random (*see sensitivity analyses in methods*). This comparison did not result in any significant differences. Figure 1 and Table S2 give an overview of the intervention use of the study sample. Information regarding the types of therapy, counseling, and medication that were used is provided in Tables S2, S4, and S5.

#### The preferred clustering-solution contained five clusters

Both the four- and the five-cluster solution contained only clusters with *n* > 10% of the sample and had the highest, but almost equal, ASWw values (Table 3; Tables S6; S7). Both cluster solutions had two clusters with ASWw values indicating a reasonable structure (ASWw = 0.51–0.70) and other clusters indicating weakly structured data (ASWw = 0.26–0.50). In addition, both solutions had one cluster with an ASWw value that approximated 0, indicating no structure in the data. Fewer participants were assigned to this specific cluster in the five-cluster solution. The JC values in the five-cluster solution indicated that all clusters had relatively good stability. Thus, given the smaller size of the weakest structured cluster, the five-cluster solution was chosen as the preferred solution.

**Table 3.** The cluster size (N), weighted average silhouette width (ASWw), Jaccard similarity (JC), and the description of the intervention trajectory for each cluster of the five-cluster solution.

Cluster	*N*	ASWw	JC	Interpretation ASWw	Interpretation JC	Description
Cluster 1	125	0.58	0.80	Reasonable structure	Stable cluster	**Least intervention**
Cluster 2	51	0.64	0.60	Reasonable structure	Clear pattern	**Mostly counseling**
Cluster 3	100	0.26	0.83	Weak structure	Stable cluster	**Mostly medication**
Cluster 4	84	0.30	0.69	Weak structure	Clear pattern	**Mixed, most C & M**
Cluster 5	85	0.01	0.78	No structure	Stable cluster	**-**

M: medication; C: counseling.

For ASWw and JC, a higher value indicates higher internal structure or stability.

**Figure 2.**
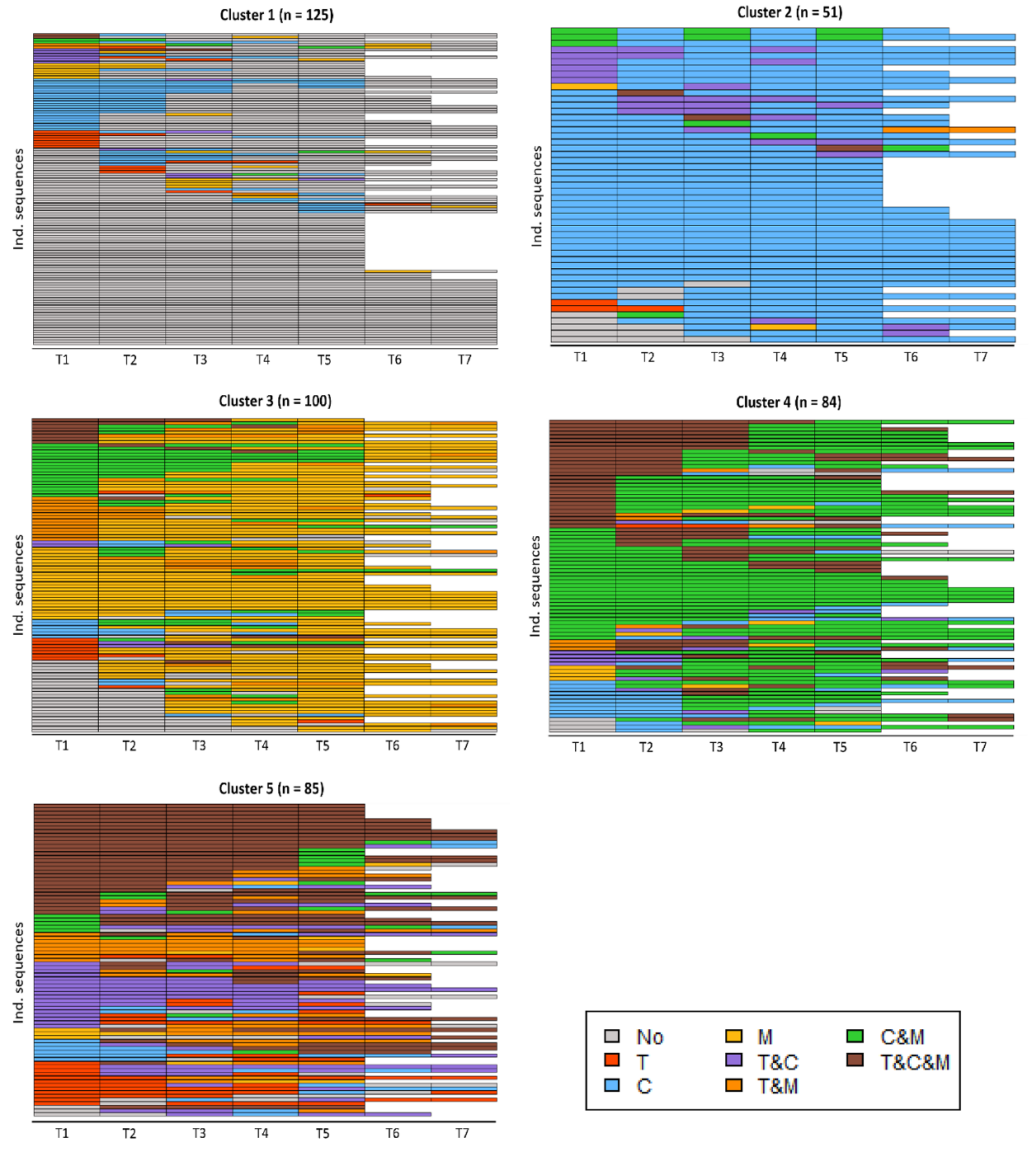
State index plots of each of the five different clusters that were obtained after clustering autistic adults based on their intervention use. The different rows in each plot visualize individual state sequences (intervention trajectories) within each cluster. The x-axis indicates time points (T1–T7). All individual sequences were left-aligned, implying that all sequences were starting at T1. Individuals with 5 or 6 years data points therefore have sequences ending at T5 or T6. T: therapy; C: counseling; M: medication.

**Figure 3.**
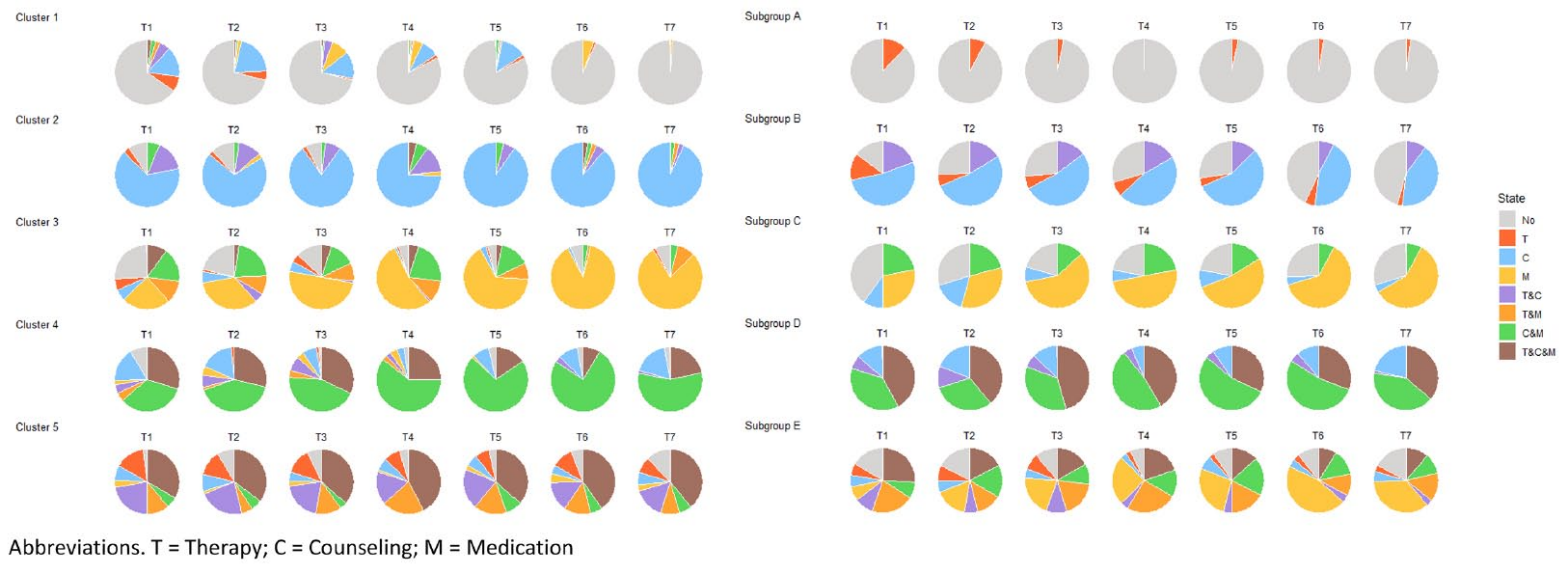
Pie charts indicating the proportion of individuals in each state for each cluster (SA) and subgroup (GBMT). T: therapy; C: counseling; M: medication.

Figure 2 shows all individual sequences for each of the five clusters. Figure 3 and Table S7 display the proportion of participants in each state for each cluster at each time point. Cluster 1 was named “least intervention,” because individuals in this cluster were most frequently in the state reflecting “no intervention use” or in the state “counseling.” Cluster 2 was named “mostly counseling,” as the vast majority of individuals in this cluster was using counseling across all time points, in some instances concurrently with either therapy or medication. Cluster 3 was named “mostly medication,” because this cluster was dominated by individuals in the state “medication,” followed by “counseling and medication.” Cluster 4 was named “mixed, mostly counseling and medication,” corresponding to the most frequently occurring states in this cluster. While there are clearly more dominant patterns in the different clusters, alternative sequences were present in each cluster as well (Figure 2). There is not a single cluster that was mostly characterized by therapy use, which means that the individuals using therapy most often used this in combination with another intervention type and were divided across different clusters. Finally, Cluster 5 had an ASWw value of 0.01, indicating this is not a well-defined cluster. Instead there is great inter-individual variability of intervention trajectories within this cluster. As such, we refrained from labeling the intervention trajectory characterizing Cluster 5. Inspecting individual trajectories within this cluster reveals heterogeneity in intervention trajectories, but that most individuals used different interventions concurrently at multiple time points, including “therapy and medication” and “therapy and counseling.” Finally, therapy use was not associated with only one cluster, which means that the individuals who most often used therapy did so in combination with another intervention type and were divided across several clusters.

#### Sensitivity analyses corroborate the cluster solution

Both sensitivity analyses resulted in a five-cluster solution, thereby corroborating results of the initial SA (Table S9; Table S10). The most dominant intervention trajectories corresponded to those that resulted from the initial analysis (Figure S2; Figure S3). Again, the ASWw value for Cluster 5 was very low, whereas the ASWw values for the other clusters were indicating a reasonable structure in the data (Table S11; Table S12). Together, the results of these sensitivity analyses strengthen the results of the initial analysis.

#### Cross-method validates four out of five clusters

A model with five subgroups best fitted the data using GBMT modeling, given that this model had the lowest BIC and AIC, while including only subgroups with a size *n* > 10% of the size of the study sample (Table S13). Visual and numerical exploration of the GBMT subgroups are described in Figure 3, Figure S5, and Table S8. Below we further describe the GBMT results (Subgroups A–E) and make a comparison to the SA results (Clusters 1–5; Figure 3; Table 4).

**Table 4.** Summary of the comparison of the SA-clustering and GBMT-subgrouping comparison.

Cluster	*N* cluster	Corresponding subgroup	*N* subgroup	Description	% of Cluster in subgroup	% of Subgroup in cluster
Cluster 1	125	Subgroup A	62	Least intervention	48.0%	96.8%
Cluster 2	51	Subgroup B	95	Mostly counseling	76,5%	41.1%
Cluster 3	100	Subgroup C	68	Mostly medication	45.0%	66.2%
Cluster 4	84	Subgroup D	94	Mixed, mostly C&M	69.0%	61.7%
Cluster 5	85	-	-	-	-	-
-		Subgroup E	126	-	-	-

C: Counseling; M: medication.

### Criteria 1 and 2: overlapping intervention trajectories between GBMT and SA

Approximately 90% of the individuals in Subgroup A were not using any intervention, thereby overlapping most with Cluster 1 (*least intervention use*). Individuals in Subgroup B mostly used counseling or did not use any intervention, therefore corresponding to Cluster 2 (*mostly counseling*). Subgroup C was dominated by individuals using medication or not using intervention, therefore this subgroup corresponds to Cluster 3 (*mostly medication*). Individuals in Subgroup D predominantly used both medication and counseling concurrently, thereby best mirroring Cluster 4 (*mixed, most counseling and medication*). Despite considerable overlap content-wise, corresponding Clusters and Subgroups differed in their size. That is, Cluster 1 and Cluster 3 were larger than their corresponding Subgroups, whereas Cluster 2 was smaller than its corresponding Subgroup (Table 4). Finally, Subgroup E was characterized by great variability in intervention use, hindering our ability to define a general pattern of intervention use. Even though this seems to mirror Cluster 5, Subgroup E did not seem to correspond well to Cluster 5 (Table S8). Together, this comparison indicated that Clusters 1–4, but not Cluster 5, had a corresponding subgroup characterized by an overlapping intervention trajectory and consisting of >50% of participants assigned to the same state on each time point.

### Criteria 3: at least 75% of the participants of cluster in corresponding subgroup

Table 4 shows the proportion of individuals in each cluster that were assigned to their corresponding subgroup. This shows that Cluster 2 (*mostly counseling*) was meeting criteria 3 and that Cluster 4 *(mixed, mostly counseling and medication*) was almost meeting criteria 3. The differences in size between Cluster 1 and Subgroup A (*least intervention*) and between Cluster 3 and Subgroup C (*mostly medication*) hamper meeting criteria 3. However, we found considerable overlap in participants between Clusters 1 and 3 and their corresponding subgroups. Of note, even though the intervention trajectory characterizing Cluster 5 was not well reflected in Subgroup E, individuals in Cluster 5 were most often assigned to this specific subgroup (43%).

#### Clusters differ in demographic characteristics

Together, our results provide support that Clusters 1–4 are meaningful clusters in our data. For this reason, we provide demographic characteristics for all clusters, but only statistically compare Clusters 1–4 (Table 5). Cluster 1 was most often different from one or more clusters. Specifically, Cluster 1 consisted of a significantly larger proportion of males and a smaller proportion of individuals with a co-occurring psychiatric condition as compared to all other clusters. In addition, individuals in Cluster 1 were significantly older than individuals in Cluster 4 (*p* = 0.040) and were diagnosed at a significantly older age than individuals in Cluster 2 (*p* = 0.038). Finally, individuals in Cluster 1 more frequently had a partner than individuals in Clusters 2 (*p* = 0.023) and Cluster 4 (*p* = 0.004). Individuals in Cluster 4 less frequently had paid employment than individuals in Cluster 1 (*p* < 0.001) and Cluster 3 (*p* < 0.001). Finally, Table S14 presents an overview of the demographic characteristics of Subgroups A–E. These results indicate that GBMT subgroups and SA clusters were characterized by a similar pattern of demographic characteristics, thereby strengthening the existence of Clusters 1–4 in the data.

**Table 5.** Demographic information and rates of co-occurring psychiatric conditions for each of the five SA clusters and statistical comparisons between clusters 1 and 4.

Characteristic	Cluster 1	Cluster 2	Cluster 3	Cluster 4	Statistics				Cluster 5
	*N* = 125	*N* = 51	*N* = 100	*N* = 84	*F*/χ^2^	*p*	*V*/ɳ^2^	Post hoc	*N* = 85
	Least intervention	Mostly C	Mostly M	Mixed C&M					
Chronological age					3.48	.016	0.03	1 > 4	
Mean in years	52.9	47.3	49.7	47.8					44.5
(SD)	(14.7)	(14.4)	(12.4)	(11.3)					(13.0)
Age of diagnosis					3.37	0.19	0.03	1 > 2	
Mean in years	40.5	33.3	37.7	35.2					33.7
(SD)	(16.2)	(15.9)	(14.5)	(13.8)					(14.7)
Time since diagnosis					0.75	0.523	0.01		
Mean in years	12.2	13.5	11.9	12.5					10.6
(SD)	5.50	5.03	5.96	6.73					(4.86)
AQ score					1.46	0.226	0.01		
Mean	82.0	82.3	84.4	84.7				-	85.52
(SD)	(11.2)	(9.92)	(10.8)	(10.2)					(10.3)
Biological sex					23.3	<0.001	0.26	1 < 2,3,4	
Female	45 (36%)	32 (63)	60 (60)	54 (64)					60 (71)
Ethnicity					1.69	0.639	0.07		
Dutch	121 (97%)	47 (94)	98 (98)	80 (96)					83 (99%)
Educational level^a^					9.47	0.148	0.13	-	
Low	4 (4%)	6 (15%)	9 (12%)	5 (8%)					6 (9%)
Medium	27 (29%)	16 (40%)	28 (36%)	23 (37%)					20 (29%)
High	61 (66%)	17 (43%)	39 (51%)	37 (55%)					41 (60%)
With partner	78 (62%)	20 (39%)	55 (55%)	32 (38%)	17.2	<0.001	0.22	1 > 2,4	34 (40%)
Paid employment	74 (59%)	23 (45%)	59 (59%)	24 (29%)	25.7	<0.001	0.28	4 < 1,3	38 (45%)
Co-occurring psychiatric condition	21 (17%)	19 (37%)	48 (48%)	52 (62%)	49.0	<0.001	0.38	1 < 2,3,4	44 (52%)
ADHD	7 (6%)	5 (10%)	18 (18%)	11 (13%)					14 (17%)
Mood disorder	6 (5%)	3 (6%)	21 (23%)	29 (35%)					22 (26%)
Anxiety disorder	5 (4%)	2 (4%)	11 (11%)	18 (21%)					10 (12%)
Psychosis spectrum	1 (1%)	0 (0%)	0 (0%)	3 (4%)					0 (0%)
Personality disorder	1 (1%)	1 (2%)	3 (3%)	12 (14%)					5 (6%)
Eating disorder	0 (0%)	4 (8%)	0 (0%)	2 (2%)					2 (2%)

C: counseling; M: medication; ADHD: attention-deficit hyperactivity disorder; *V*: Cramer’s *V*.

a The reported frequencies do not add up to 100%, because some individuals answered “other” and were not included in the calculation.

## Discussion

Autistic adults make frequent use of mental health interventions (Weiss et al., 2018; Zerbo et al., 2019), including therapy, counseling, and medication. However, much remains unknown concerning the longitudinal and concurrent use of different kinds of interventions. In addition, inter-individual differences in intervention use are not well understood. This is partly due to the inherent heterogeneity in earlier samples, which included not only both children and adults but also autistic people with and without an intellectual disability. Both developmental stage and intellectual ability are likely to impact intervention use. To address these issues, this study investigated the intervention trajectories across 5–7 years in a Dutch community sample of 445 autistic adults without an intellectual disability by means of sequence analysis. This led to the identification of four coherent and stable clusters of autistic adults, each characterized by a distinct intervention trajectory (*least intervention, mostly counseling, mostly medication, mixed counseling & medication*). These clusters differed on various aspects, most noteworthy on the proportion of females and the rate of co-occurring psychiatric conditions.

Overall, counseling was the intervention type that was used most frequently by the autistic adults (~50% of individuals), followed by medication (~45%), and therapy (~25%). These rates of medication and counseling use fall within the range that was reported in previous studies (e.g. Ishler et al., 2022; Jobski et al., 2017), whereas the self-reported rate of therapy was relatively low (e.g. Schott et al., 2021; Vogan et al., 2017). However, our results cannot be directly compared to earlier studies, because in contrast to these studies, we studied a relatively homogeneous sample that consisted of only autistic adults without an intellectual disability. Therefore, our results offer new insights on intervention use in autistic adults without an intellectual disability. Specifically, we observed that also the majority of autistic individuals without intellectual disability frequently used mental health care services, implying that this group requires attention from both a clinical and research perspective.

By detecting four distinct clusters in our data, this study sheds light on the diversity of intervention use among autistic adults. The first cluster (“*least intervention*”) contained the autistic adults with minimal-to-no-intervention use. It may be that individuals in this cluster experienced barriers to accessing care and therefore did not use interventions (Malik-Soni et al., 2022). Alternatively, our data suggest that the individuals in this cluster may not require much intervention. That is, this cluster had the highest rates of individuals with paid employment and a partner, and the lowest rates of co-occurring psychiatric conditions, which were comparable to the general population (Lai et al., 2019). The second cluster was characterized by counseling use as primary intervention type (“mostly counseling”). Although not significantly different, individuals in this cluster were less frequently employed and more often reported a lower educational level than individuals in the first cluster, suggesting that counseling provided assistance with vocational or practical matters. Individuals in the third cluster (“mostly medication”) were predominantly using medication for the entire study period. The proportion of individuals with a co-occurring psychiatric condition was larger in this cluster as compared to the first two clusters, which aligns with the intended goal of medication use, to mostly treat co-occurring mental health problems (Jobski et al., 2017). The intervention trajectory of individuals in the fourth subgroup (“mixed, mostly C&M”) was characterized by using counseling and medication concurrently for the entire study period. There were relatively many individuals in this cluster using all three kinds of intervention simultaneously. Individuals in this cluster had the lowest employment rates and a high prevalence of co-occurring psychiatric problems, which likely explains their more intensive use of these interventions (Fusar-Poli et al., 2019; Jonkman et al., 2023).

Of additional interest is that the fourth and fifth clusters, both mostly consisting of multi-intervention users, contained the largest proportion of females. It may be that autistic females, just like females in the general population, are more inclined to seek health care (Thompson et al., 2016). In addition, our panel of clinician experts suggested that autistic females may be offered more mental health care, subsequently leading to increased rates of intervention use. This aligns with suggestions that implicit gender biases exist among health care professionals (Dijksterhuis et al., 2021; FitzGerald & Hurst, 2017), resulting in females receiving different kinds of care than males. Alternatively, our results support findings that autistic females have more co-occurring mental health problems than autistic males (Jadav & Bal, 2022; Rødgaard et al., 2021), which is reflected in increased rates of autistic females using mental health services (Tint et al., 2023).

It is plausible that interventions were, at least partly, targeting the co-occurring psychiatric problems that our participants reported. Hence, our findings seem to corroborate the results of the only previous study addressing longitudinal mental health development in autistic adults (Roestorf et al., 2022), that mental health problems in autistic individuals are likely to persist, even when individuals are using mental health care services. We did not detect a different clustering result when we excluded the COVID-19 pandemic years. This may indicate that autistic adults in this study were able to continue care, despite COVID restrictions. It may also be that those individuals who experienced difficulties accessing appropriate care were not included in our analyses, as a result of our decision to not include individuals with consecutive missing data points, whereas missing data points could be associated with experiencing more mental health problems.

### Implications and future directions

The majority of autistic adults in this community sample, especially those with co-occurring mental health conditions, used interventions for a prolonged period of time. At the same time, many individuals were employed while using a form of intervention. These observations underscore that access to mental health care for autistic adults throughout the lifespan is key, to promote both their well-being and societal participation (Maddox et al., 2021; Oakley et al., 2021). In addition many autistic adults used different kinds of interventions concurrently, which poses a challenge for care-coordination, involving multiple professionals and service systems. To address this challenge, multidisciplinary collaboration in clinical care is crucial, for instance by co-designing the care pathway with the autistic individual themselves, their psychologist and counselor (Lord et al., 2022; Matson et al., 2016). In addition, life coaches supporting autistic individuals in multiple domains might provide assistance in managing the care process and as such enable tailored mental health care across adulthood (Vanuit Autisme Bekeken, 2019).

Our results seem to suggest that our clustering method grouped the individuals with atypical trajectories together in Cluster 5, which may explain the low statistical specificity of this cluster. Strikingly, despite its statistical insignificance, inspection of the data reveals that this may be a cluster of clinical significance. Specifically, the individuals in Cluster 5 were characterized by great heterogeneity in intervention use and by multi-intervention use, suggesting that these individuals may actually be those in high need of clinical attention and support. As this cluster was characterized by high rates of co-occurring mental health problems and included many female participants, this further signals the complexity of designing adequate intervention approaches for autistic adults, especially for female adults or those with co-occurring psychiatric conditions.

Our data on real-life intervention use has important clinical implications. For future studies, it would be of interest to include complementary information on autistic adults’ experiences regarding accessing mental health services and intervention outcomes. This allows to study to what extent real-life intervention use adequately reflects the needs of autistic adults and to pinpoint areas for mental health service improvement (Chan & Doran, 2024; Mazurek et al., 2023).

A better understanding of experienced preferences or barriers of intervention use improves our ability to tailor the intervention approach to the needs of autistic adults. Based on our results, we recommend future studies on the potentially protective role of being employed or having a partner relationship, as this characterized the cluster with the least intervention use. Of additional interest are the relatively low rates of therapy use that were found in the present study. It may be that autistic adults prefer using medication over talking therapy (Maddox et al., 2018), which seems supported by our, and previous, findings that autistic adults used medication without an associated comorbid diagnosis (Feroe et al., 2021). Furthermore, it would be of interest to study which elements of counseling, the most frequently used type of intervention, contribute most to the well-being of autistic adults (Chan & Doran, 2024). Moreover, our findings highlight that studies are needed on how to best address co-occurring mental health problems and the needs of autistic females. While progress has been made (Leedham et al., 2020; Rosen et al., 2021; White et al., 2018), we reiterate the call for prioritizing this on the research agenda (Benevides et al., 2020).

### Strengths and limitations

Alongside its strengths, including the use of a community sample with a large sample size and longitudinal data analysis, this study has some limitations. First, our sample consisted of a relatively large proportion of females, highly educated individuals with an adult diagnosis. Moreover, our study sample included a slightly lower proportion of individuals with a co-occurring mental health condition than our total sample, which may limit our generalizability. Moreover, our participants provided limited information about the impact of their co-occurring condition. More detailed information regarding the duration of such co-occurring conditions and to what extent specific co-occurring conditions are related to receiving specific types of interventions is an important next step. Generalizability may also be limited, because the interventions that were offered to autistic adults in the Netherlands might differ from other countries. In line with previous suggestions from the United Kingdom (Houghton et al., 2018), our results may indicate that medication use is lower among Dutch autistic individuals than in North-American countries, underscoring the importance of considering differences in cultural settings in understanding intervention use of autistic adults.

The SA result indicated that only Clusters 1 (“least intervention”) and 2 (“mostly counseling”) were internally coherent and could be clearly distinguished from other clusters. It was a strength of our study that we employed two additional sensitivity analyses and cross-method validation (Agelink van Rentergem et al., 2021), all resulting in a highly similar clustering result. Sequence analysis is a relatively new method to investigate care trajectories (e.g. Roth et al., 2022; Roux et al., 2019). As compared to other approaches (e.g. GBMT), SA allows to directly observe individual intervention trajectories. In addition, variation within different subgroups can be observed. Together, this enhanced our understanding of longitudinal patterns of intervention use of autistic adults. Here, it is important to keep in mind that we constructed intervention trajectories based on a yearly measure of intervention use with a binary outcome. Alternatively, intervention trajectories could be used to investigate stability and change in different types of therapy, medication, or counseling or in the intensity of intervention use. Clustering such trajectories may be of interest for follow-up research, thereby potentially also enabling further insight into the re-use of interventions by autistic adults.

## Conclusion

The majority of autistic individuals in this community sample used a form of mental health support for a prolonged period of time, but these adults also varied greatly from each other in their intervention use. Having a co-occurring psychiatric condition and being female was associated with increased intervention use, indicating that awareness of, and further research addressing, the needs of these individuals in particular is warranted. Autistic adults may rely on interventions to improve their well-being, but also to accommodate their successful participation in society. Together, this highlights that accessible and suitable mental health support throughout the lifespan for autistic adults is not only beneficial to improve individual functioning, but also for society as a whole.

## Supplemental Material

sj-docx-1-aut-10.1177_13623613241304513 – Supplemental material for Mental health care use of autistic adults: Identifying longitudinal patterns using sequence analysis Supplemental material, sj-docx-1-aut-10.1177_13623613241304513 for Mental health care use of autistic adults: Identifying longitudinal patterns using sequence analysis by Iris Selten, Tim Ziermans, Iris Rapoport, Kim Jonkman and Hilde M Geurts in Autism

## Notes

1. We chose to use identity-first terminology consistently throughout the article; however, we are aware of the variation within the autism community regarding preferences for person-first and identity-first terminology (e.g. Bosman & Thijs, 2023; Buijsman et al., 2023; Geelhand et al., 2023; Taboas et al., 2023).

2. In the Dutch health care system, expenses related to therapy and mediation are covered via the individual’s mandatory health care insurance, whereas counseling is covered via the municipality.

3. Participants were first requested to indicate therapy use, using the following question: Did you use psychological therapy this year or since you filled out the previous questionnaire? N.B. This specifically concerns therapeutic intervention, not counseling. Counseling will be discussed later in the questionnaire. Subsequently, participants were requested to indicate counseling use as follows: Have you had any guidance or coaching related to your autism in the past year? N.B. This includes guidance in living, working, school, and studying that takes place both at home and outside of the home situation.
