# Supplementary material for: Mental health care use of autistic adults: Identifying longitudinal patterns using sequence analysis
Source: Autism. 2024 Dec 18;29(6):1431–45. doi: 10.1177/13623613241304513 (PMC12089683; doi:10.1177/13623613241304513)
Supplement: sj-docx-1-aut-10.1177_13623613241304513 – Supplemental material for Mental health care use of autistic adults: Identifying longitudinal patterns using sequence analysis [file sj-docx-1-aut-10.1177_13623613241304513.docx]

Supplementary materials

Figure S1. Flowchart to illustrate the different steps that were taken to handle missing datapoints


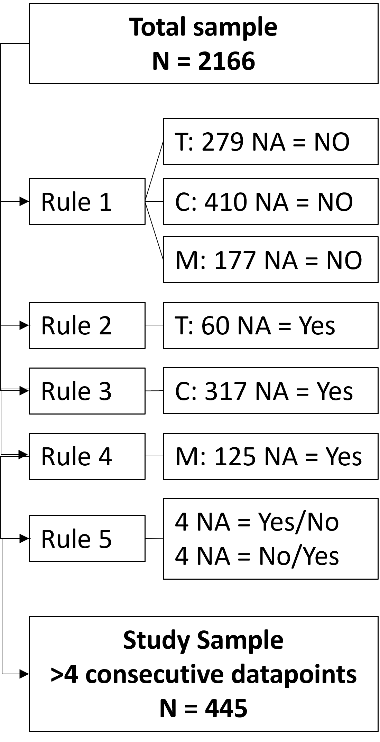


Abbreviations. T = therapy; C = counseling; M = medication; NA = missing datapoint

Explanation to the figure

Intervention use was measured each year with the following question with binary outcome (yes/no): “*Did you use psychosocial intervention/pharmacological intervention/counselling in the past year, or since the last time you filled out this questionnaire?”.* Participants additionally provided the duration of psychosocial intervention.

To fill in missing datapoints (“NA”) for all individuals in the Total Sample (N=2166), we specified five rules that were only applied in case of a single consecutive missing datapoint.
*Rule 1*. Missing datapoints on therapy, counseling and medication which were followed by the answer "no" on the subsequent measurement were filled in with "no".
*Rule 2*. Missing datapoints for medication that were surrounded by two datapoints indicating "yes" and which both report the same type of medication were filled in with "yes". Otherwise, the missing datapoint remained coded as "NA".
*Rule 3*. Missing datapoints for counselling which were surrounded by two datapoints indicating "yes" were filled in with "yes". Otherwise, the missing datapoint remained coded as "NA".
*Rule 4*. Missing datapoints for therapy which were followed by the answer "yes, with a duration of more than 7 months" on the subsequent measurement were filled in with "yes".
*Rule 5*. After step 4, we calculated the number of participants with at least 5 consecutive available datapoints on *therapy* after completing rules 1-4 (N = 448). A total of 8 participants had missing datapoints on therapy followed by the answer "yes, but with a duration of less than 7 months" on the subsequent measurement. This meant that the proportion of participants with such missing data is smaller than 5% of the Subsample. In line with our preregistration, therefore, for half of these participants the missing datapoint were filled in as "no" and the subsequent datapoint as "yes". For the other half of these participants, we the opposite order of yes and no was used.

Computation of study sample.
Participants with at least 5 consecutive available datapoints on all three measures of intervention use (i.e. *therapy, counseling and medication*) were included in data analysis (N=445).

|  | Study sample before | | | Study sample after | | |
| --- | --- | --- | --- | --- | --- | --- |
|  | Therapy | Counseling | Medication | Therapy | Counseling | Medication |
| 2016 | 134 | 134 | 134 | 133 | 139 | 135 |
| 2017 | 74 | 74 | 74 | 67 | 65 | 70 |
| 2018 | 9 | 9 | 9 | 0 | 0 | 0 |
| 2019 | 11 | 11 | 13 | 0 | 0 | 0 |
| 2020 | 0 | 0 | 1 | 0 | 0 | 0 |
| 2021 | 38 | 445 | 46 | 27 | 88 | 25 |

Table S1. Number of missing values in the study sample per year before and after applying our procedure for filling missing datapoint (N=445).

Note. Missing datapoints in the years 2015 and 2022 could not be filled, as we used information of the surrounding years to fill in missing values.

Table S2. Proportion of individuals with each type and combination of intervention use for each year for the study sample (N=445).

| **State** | **2015** | **2016** | **2017** | **2018** | **2019** | **2020** | **2021** |
| --- | --- | --- | --- | --- | --- | --- | --- |
|  | N=296 | N=373 | N=445 | N=445 | N=445 | N=354 | N=350 |
| T&C&M | 13.9% | 12.6% | 14.4% | 14.4% | 10.6% | 9.6% | 11.4% |
| T&C | 8.1% | 7.8% | 7.4% | 5.6% | 4.7% | 4.0% | 3.4% |
| C&M | 11.8% | 13.9% | 12.4% | 17.3% | 19.3% | 16.9% | 13.4% |
| T&M | 6.1% | 3.5% | 5.2% | 7.2% | 4.9% | 3.1% | 4.0% |
| T | 6.8% | 4.6% | 4.0% | 2.2% | 2.0% | 2.3% | 1.7% |
| C | 17.2% | 19.8% | 16.4% | 13.0% | 17.3% | 14.1% | 16.3% |
| M | 6.8% | 9.7% | 14.8% | 14.6% | 15.7% | 22.0% | 18.6% |
| No | 29.4% | 28.2% | 25.4% | 25.6% | 25.4% | 28.0% | 31.1% |

*Abbreviations: T = therapy, C = counseling, M = medication*

Table S3. The proportion of participants in the study sample using each type of therapy for the top 5 most frequently used types of therapy.

| **Type of therapy** | **Proportion** |
| --- | --- |
| Individual therapy |  |
| 1 year | 17.1% |
| 2 years | 11.2% |
| >2 years | 20.0% |
| Psycho-education |  |
| 1 year | 13.26% |
| >1 year | 3.14% |
| Mindfulness therapy |  |
| 1 year | 10.6% |
| >1 year | 3.14% |
| Conversation group |  |
| 1 year | 9.66% |
| >1 year | 2.02% |
| Motor therapy |  |
| 1 year | 8.31% |
| >1 year | 5.39% |

*Note. Individual therapy is defined as regular one-on-one sessions with psychologist/psychiatrist
 The total number of years that participants used a specific type of therapy was calculated, therefore >1 or >2 years does not automatically indicate consecutive years.*

Table S4. Proportion of participants in the study sample using medication for the 4 categories of medication that were measured in the NAR

| **Type of therapy** | **Proportion** |
| --- | --- |
| Stimulants |  |
| 1-2 years | 4.5% |
| >2 years | 8.31% |
| Anti-depressants |  |
| 1-2 years | 12.1% |
| >2 years | 28.5% |
| Anxiolytics |  |
| 1-2 years | 11.9% |
| >2 years | 10.1% |
| Anti-psychotics |  |
| 1-2 years | 7.64% |
| >2 years | 20.5% |

*Note. The total number of years that participants used a specific type of medication was calculated, therefore >1 or >2 years does not automatically indicate sequential years.*

Table S5. Proportion of participants in the study sample using each type of counseling for the top 5 types of counseling

| **Type of counseling** | **Proportion** |
| --- | --- |
| Personal development |  |
| 1-2 years | 27.6% |
| >2 years | 31.7% |
| Social skills |  |
| 1-2 years | 25.9% |
| >2 years | 27.7% |
| Free time |  |
| 1-2 years | 22.2% |
| >2 years | 16.9% |
| Living skills |  |
| 1-2 years | 18.0% |
| >2 years | 20.9%‬ |
| Job support |  |
| 1-2 years | 19.8% |
| >2 years | 21.6‬% |

*Note. The total number of years that participants used a specific type of medication was calculated, therefore >1 or >2 years does not automatically indicate sequential years.*

Table S6. The Weighted Average Silhouette Width (ASWw), range of Jaccard Similarity coefficients (JC) and sizes of the clusters of the different cluster solutions.

| **Solution** | **ASWw** | **JC-range** | **Cluster size** |
| --- | --- | --- | --- |
| 2 clusters | 0.26 | 0.88-0.94 | 125; 320 |
| 3 clusters | 0.3 | 0.54-0.87 | 125; 100; 220 |
| 4 clusters | 0.34 | 0.76-0.87 | 125; 51; 100; 169 |
| 5 clusters | 0.35 | 0.58-0.81 | 125 ; 51 ; 100; 84; 85 |
| 6 clusters | 0.37 | 0.63-0.83 | 125 ; 51 ; 100 ; 84 ; 50 ; 35 |

| **4 clusters** | **N** | **ASWw** | **JC** |
| --- | --- | --- | --- |
| Cluster 1 | 125 | 0,58 | 0.81 |
| Cluster 2 | 51 | 0,65 | 0.76 |
| Cluster 3 | 100 | 0,31 | 0.79 |
| Cluster 4 | 169 | 0,09 | 0.87 |

Table S7. The size (N), the Weighted Average Silhouette Width (ASWw) and the Jaccard Similarity coefficients (JC) of each cluster in the four-cluster solution

Table S8. Percentages of therapy, counseling and medication use for each cluster from the GBMT solution and their corresponding SA cluster. Percentages in **bold** indicate the most frequently occurring state and percentages in *italics* the second most frequently occurring state at each timepoint (T1-T7).

| **Type of intervention use** | **Least intervention** | | **Mostly counseling** | | **Mostly medication** | | **Mixed mostly C&M** | | **-** | **-** |
| --- | --- | --- | --- | --- | --- | --- | --- | --- | --- | --- |
|  | Cluster 1 | Subgroup A | Cluster 2 | Subgroup B | Cluster 3 | Subgroup C | Cluster 4 | Subgroup D | Cluster 5 | Subgroup E |
|  | N = 125 | N = 62 | N = 51 | N = 95 | N = 100 | N = 68 | N = 84 | N = 94 | N = 85 | N = 126 |
| **T1** |  |  |  |  |  |  |  |  |  |  |
| T&C&M | 2.2 | 0.0 | 0.0 | 0.0 | 10.0 | 0.0 | *29.8* | **42.2** | **33.3** | **25.9** |
| T&C | 5.4 | 0.0 | *15.6* | *19.4* | 0.0 | 0.0 | 4.3 | 6.7 | *22.2* | 9.4 |
| C&M | 2.2 | 0.0 | 6.3 | 0.0 | 17.1 | 22.0 | **34.0** | *37.8* | 5.6 | 8.2 |
| T&M | 2.2 | 0.0 | 0.0 | 0.0 | 11.4 | 0.0 | 4.3 | 0.0 | 11.1 | 21.2 |
| T | 7.5 | *12.2* | 3.1 | 13.4 | 5.7 | 0.0 | 0.0 | 0.0 | 14.8 | 5.9 |
| C | *15.1* | 0.0 | **65.6** | **52.2** | 5.7 | 10.0 | 17.0 | 13.3 | 7.4 | 5.9 |
| M | 0.0 | 0.0 | 0.0 | 0.0 | *24.3* | *28.0* | 2.1 | 0.0 | 3.7 | 7.1 |
| No | **65.6** | **87.8** | 9.4 | 14.9 | **25.7** | **40.0** | 8.5 | 0.0 | 1.9 | *16.5* |
| **T2** |  |  |  |  |  |  |  |  |  |  |
| T&C&M | 0.9 | 0.0 | 0.0 | 0.0 | 2.3 | 0.0 | *28.8* | **39.2** | **35.2** | **17.3** |
| T&C | 0.0 | 0.0 | *11.6* | 16.3 | 4.7 | 0.0 | 6.1 | 10.8 | *22.5* | 6.7 |
| C&M | 0.9 | 0.0 | 2.3 | 0.0 | *22.1* | 21.1 | **40.9** | *31.1* | 5.6 | *16.3* |
| T&M | 0.0 | 0.0 | 0.0 | 0.0 | 9.3 | 0.0 | 1.5 | 0.0 | 5.6 | 12.5 |
| T | 4.7 | *7.7* | 2.3 | 5.8 | 1.2 | 0.0 | 1.5 | 0.0 | 12.7 | 7.7 |
| C | *20.6* | 0.0 | **69.8** | **52.3** | 5.8 | 15.8 | 16.7 | 18.9 | 8.5 | 5.8 |
| M | 1.9 | 0.0 | 2.3 | 0.0 | **33.7** | **33.3** | 4.5 | 0.0 | 1.4 | *16.3* |
| No | **71.0** | **92.3** | *11.6* | *25.6* | 20.9 | *29.8* | 0.0 | 0.0 | 8.5 | **17.3** |
| **T3** |  |  |  |  |  |  |  |  |  |  |
| T&C&M | 0.8 | 0.0 | 0.0 | 0.0 | 5.0 | 0.0 | *32.1* | **45.7** | **36.5** | 16.7 |
| T&C | 4.0 | 0.0 | *7.8* | 14.7 | 1.0 | 0.0 | 7.1 | 6.4 | *20.0* | 10.3 |
| C&M | 0.8 | 0.0 | 2.0 | 0.0 | *13.0* | 13.2 | **44.0** | *35.1* | 3.5 | 10.3 |
| T&M | 0.0 | 0.0 | 0.0 | 0.0 | 9.0 | 0.0 | 3.6 | 0.0 | 12.9 | *18.3* |
| T | 0.8 | *3.2* | 2.0 | 6.3 | 4.0 | 0.0 | 1.2 | 0.0 | 12.9 | 7.9 |
| C | *13.6* | 0.0 | **80.4** | **52.6** | 5.0 | 7.4 | 7.1 | 12.8 | 4.7 | 4.8 |
| M | 8.8 | 0.0 | 0.0 | 0.0 | **50.0** | **58.8** | 3.6 | 0.0 | 2.4 | **20.6** |
| No | **71.2** | **96.8** | *7.8* | *26.3* | *13.0* | *20.6* | 1.2 | 0.0 | 7.1 | 11.1 |
| **T4** |  |  |  |  |  |  |  |  |  |  |
| T&C&M | 0.0 | 0.0 | 3.9 | 0.0 | 5.0 | 0.0 | *25.0* | *41.5* | **42.4** | 19.8 |
| T&C | 0.8 | 0.0 | *13.7* | 16.8 | 1.0 | 0.0 | 2.4 | 4.3 | 16.5 | 4.0 |
| C&M | 0.8 | 0.0 | 5.9 | 0.0 | *22.0* | *22.1* | **60.7** | **47.9** | 0.0 | 13.5 |
| T&M | 0.8 | 0.0 | 0.0 | 0.0 | 11.0 | 0.0 | 2.4 | 0.0 | *21.2* | **25.4** |
| T | 1.6 | 0.0 | 0.0 | 7.4 | 1.0 | 0.0 | 0.0 | 0.0 | 8.2 | 2.4 |
| C | *8.8* | 0.0 | **74.5** | **46.3** | 1.0 | 5.9 | 3.6 | 6.4 | 5.9 | 3.2 |
| M | 4.8 | 0.0 | 2.0 | 0.0 | **54.0** | **50.0** | 3.6 | 0.0 | 1.2 | 24.6 |
| No | **82.4** | **100.0** | 0.0 | *29.5* | 5.0 | *22.1* | 2.4 | 0.0 | 4.7 | 7.1 |
| **T5** |  |  |  |  |  |  |  |  |  |  |
| T&C&M | 0.0 | 0.0 | 0.0 | 0.0 | 3.0 | 0.0 | *15.5* | *31.9* | **36.5** | 13.5 |
| T&C | 0.8 | 0.0 | *5.9* | 12.6 | 0.0 | 0.0 | 0.0 | 4.3 | 20.0 | 4.0 |
| C&M | 1.6 | 0.0 | 3.9 | 0.0 | *15.0* | 16.2 | **71.4** | **54.3** | 8.2 | *19.0* |
| T&M | 0.0 | 0.0 | 0.0 | 0.0 | 8.0 | 0.0 | 0.0 | 0.0 | *16.5* | 17.5 |
| T | 1.6 | *3.2* | 0.0 | 4.2 | 1.0 | 0.0 | 0.0 | 0.0 | 7.1 | 2.4 |
| C | *12.8* | 0.0 | **90.2** | **55.8** | 3.0 | 8.8 | 8.3 | 9.6 | 5.9 | 7.1 |
| M | 0.8 | 0.0 | 0.0 | 0.0 | **66.0** | **52.9** | 1.2 | 0.0 | 2.4 | **27.0** |
| No | **82.4** | **96.8** | 0.0 | *27.4* | 4.0 | *22.1* | 3.6 | 0.0 | 3.5 | 9.5 |
| **T6** |  |  |  |  |  |  |  |  |  |  |
| T&C&M | 0.0 | 0.0 | 2.3 | 0.0 | 0.0 | 0.0 | 8.6 | *30.9* | **40.3** | 8.9 |
| T&C | 0.0 | 0.0 | *4.5* | 7.6 | 0.0 | 0.0 | 2.9 | 4.9 | *14.9* | 4.0 |
| C&M | 0.0 | 0.0 | 2.3 | 0.0 | 2.5 | 7.8 | **75.7** | **53.1** | 6.0 | *12.9* |
| T&M | 0.0 | 0.0 | 2.3 | 0.0 | 1.3 | 0.0 | 0.0 | 0.0 | 13.4 | 10.9 |
| T | 1.1 | *2.4* | 0.0 | 5.1 | 0.0 | 0.0 | 0.0 | 0.0 | 10.4 | 3.0 |
| C | 0.0 | 0.0 | **88.6** | **44.3** | 1.3 | 3.9 | *10.0* | 11.1 | 4.5 | 4.0 |
| M | *5.3* | 0.0 | 0.0 | 0.0 | **88.6** | **62.7** | 0.0 | 0.0 | 4.5 | **45.5** |
| No | **93.6** | **97.6** | 0.0 | *43.0* | *6.3* | *25.5* | 2.9 | 0.0 | 6.0 | 10.9 |
| **T7** |  |  |  |  |  |  |  |  |  |  |
| T&C&M | 0.0 | 0.0 | 0.0 | 0.0 | 0.0 | 0.0 | *21.7* | *36.3* | **39.1** | 11.2 |
| T&C | 0.0 | 0.0 | *2.3* | 10.1 | 0.0 | 0.0 | 1.4 | 1.3 | *15.6* | 3.1 |
| C&M | 0.0 | 0.0 | *2.3* | 0.0 | 3.8 | 7.8 | **56.5** | **41.3** | 6.3 | 10.2 |
| T&M | 0.0 | 0.0 | *2.3* | 0.0 | *8.9* | 0.0 | 0.0 | 0.0 | 9.4 | 14.3 |
| T | 0.0 | *2.4* | 0.0 | 2.5 | 1.3 | 0.0 | 0.0 | 0.0 | 7.8 | 3.1 |
| C | 0.0 | 0.0 | **93.2** | *41.8* | 0.0 | 3.9 | 17.4 | 21.3 | 6.3 | 5.1 |
| M | **1.1** | 0.0 | 0.0 | 0.0 | **78.5** | **58.8** | 0.0 | 0.0 | 3.1 | **35.7** |
| No | **98.9** | **97.6** | 0.0 | **45.6** | 7.6 | *29.4* | 2.9 | 0.0 | 12.5 | *17.3* |

Abbreviations. T = therapy; M = medication; C = counseling

Table S9. The Weighted Average Silhouette Width (ASWw) and sizes of the clusters of the different cluster solutions that were compared in the sensitivity analysis excluding the COVID-19 years.

| **solution** | **ASWw** | **Cluster size** |
| --- | --- | --- |
| 2 clusters | 0,26 | 147, 298 |
| 3 clusters | 0,30 | 147, 66, 232 |
| 4 clusters | 0,34 | 147, 66, 59, 173 |
| 5 clusters | 0,35 | 147, 66, 59, 58, 115 |
| 6clusters | 0,38 | 147, 66, 59, 58, 75, 40 |

Table S10. The Weighted Average Silhouette Width (ASWw), and sizes of the clusters of the different cluster solutions that were compared in the sensitivity analysis in which final missing datapoints were coded as a separate state.

| **solution** | **ASW** | **Cluster size** |
| --- | --- | --- |
| 2 clusters | 0.26 | 143,302 |
| 3 clusters | 0.30 | 143, 79, 223 |
| 4 clusters | 0.33 | 143, 79, 67, 156 |
| 5 clusters | 0.36 | 143, 67, 79, 82, 74 |
| 6 clusters | 0.37 | 114, 67, 79, 82, 33, 41 |

Figure S2. Results of the sensitivity analysis to investigate clustering results without Covid years (2020/2021)


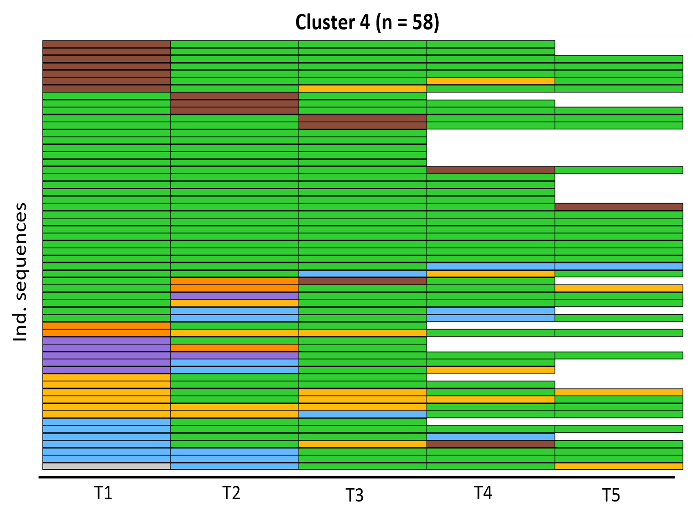

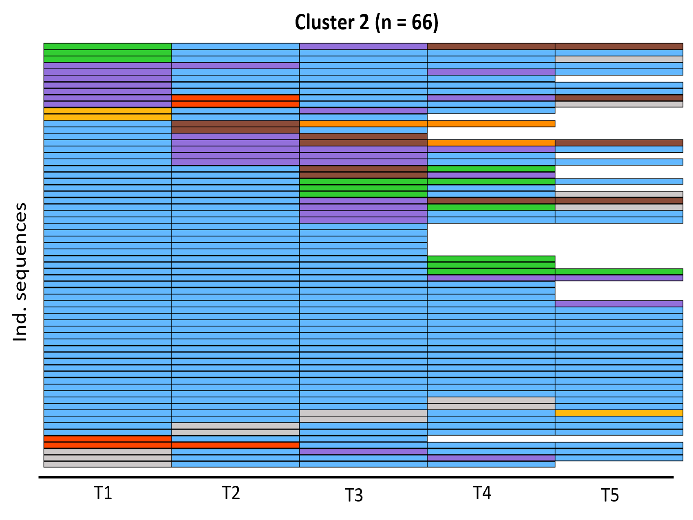


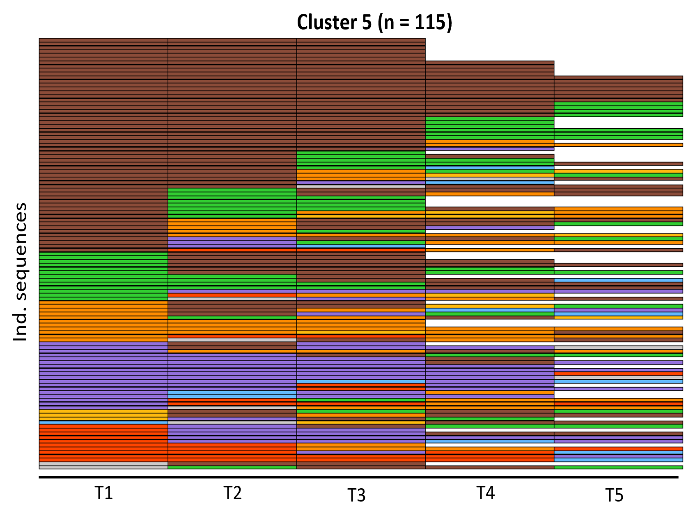

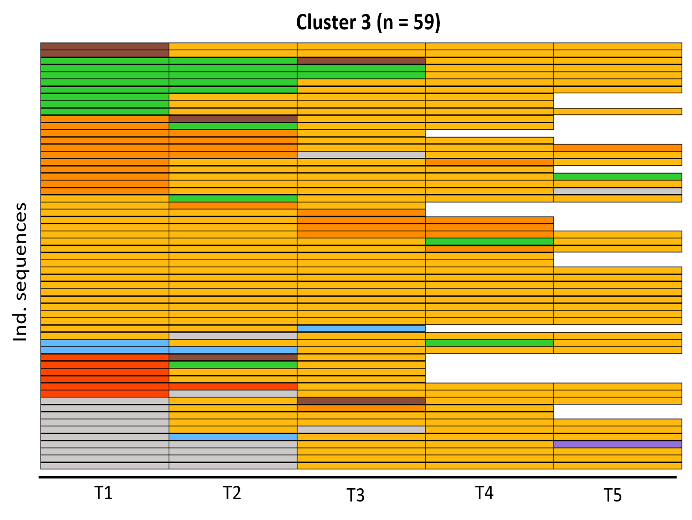

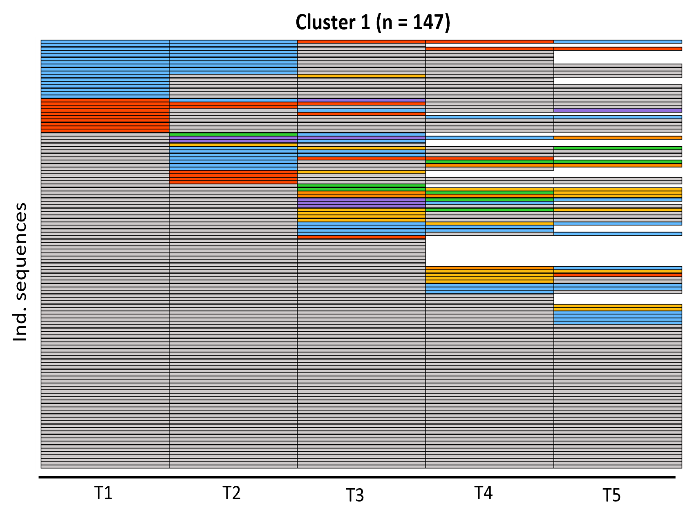


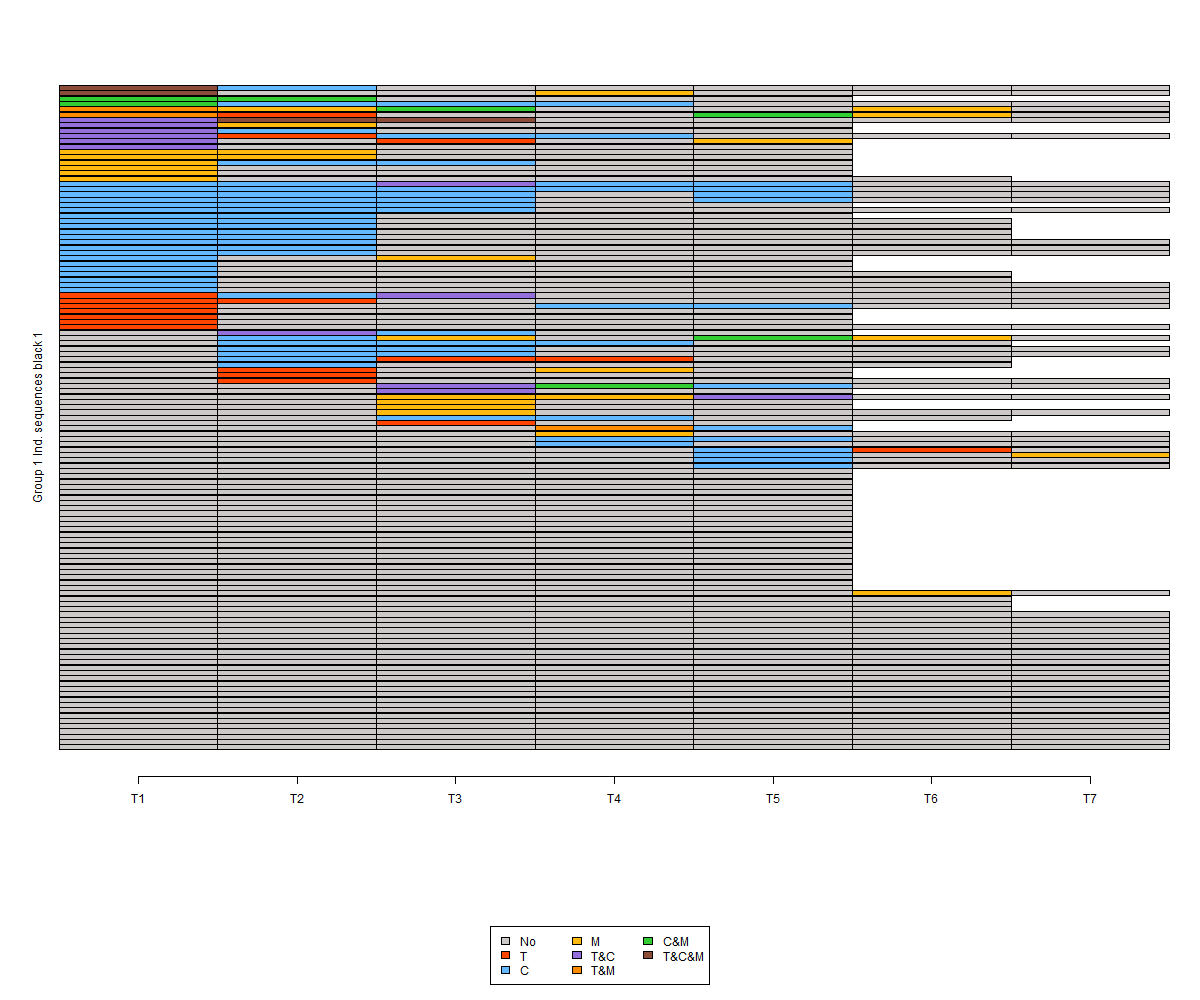


Abbreviations. T = therapy; M = medication; C = counseling


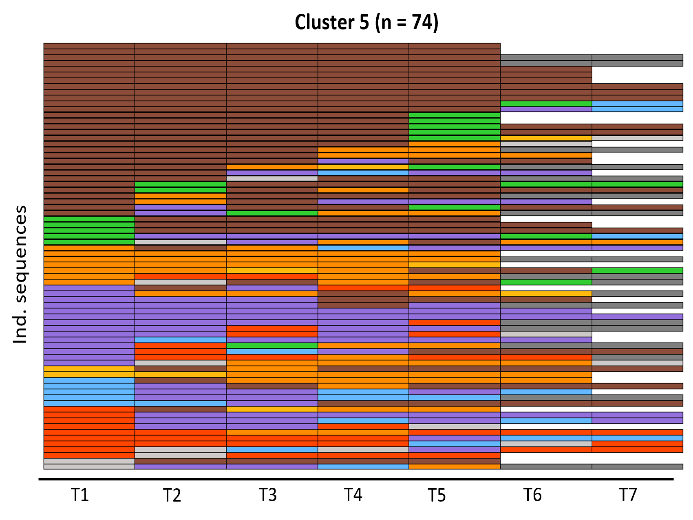

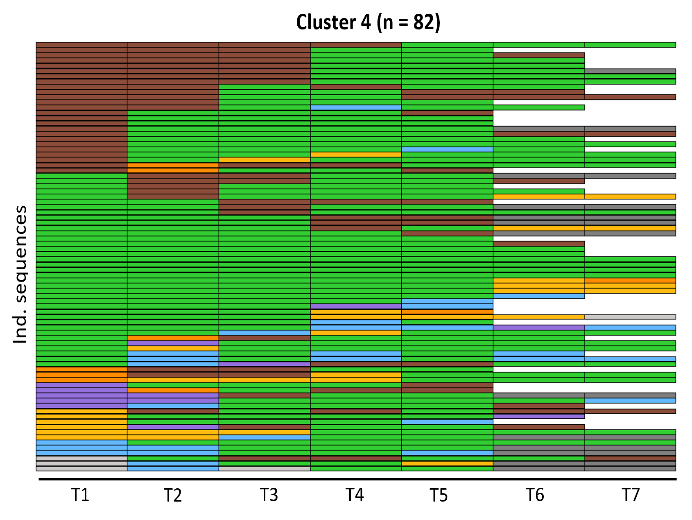

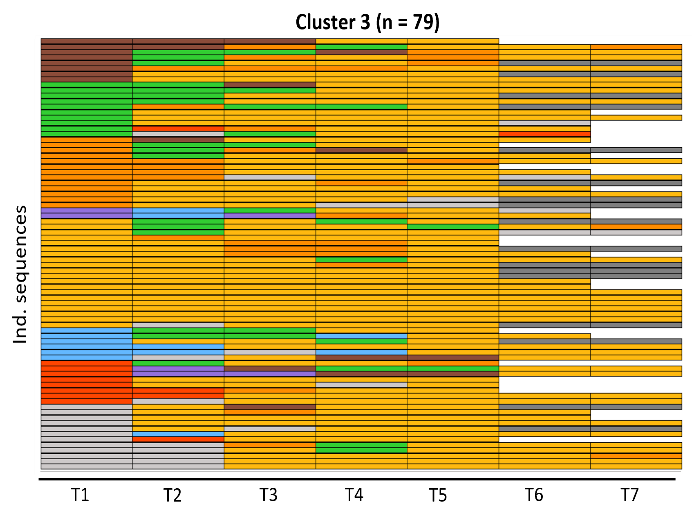

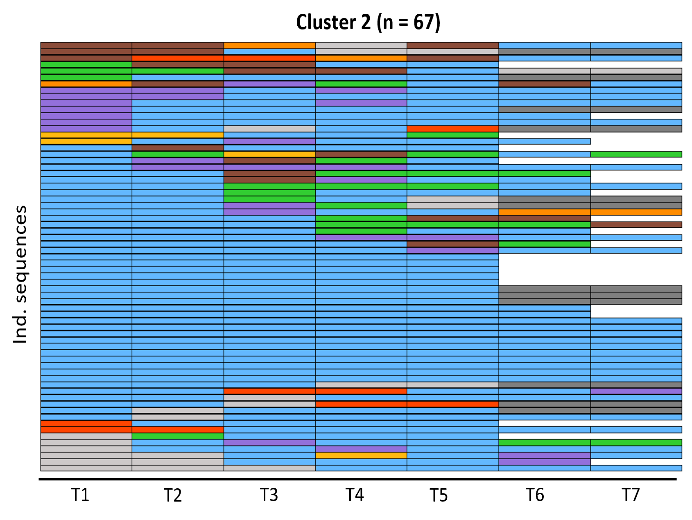
Figure S3. Sensitivity with final missing datapoints explicitly coded as separate state *(“miss”)*


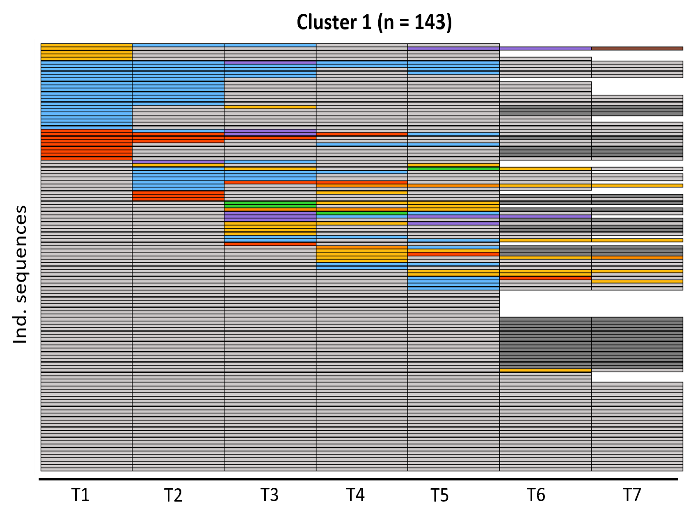


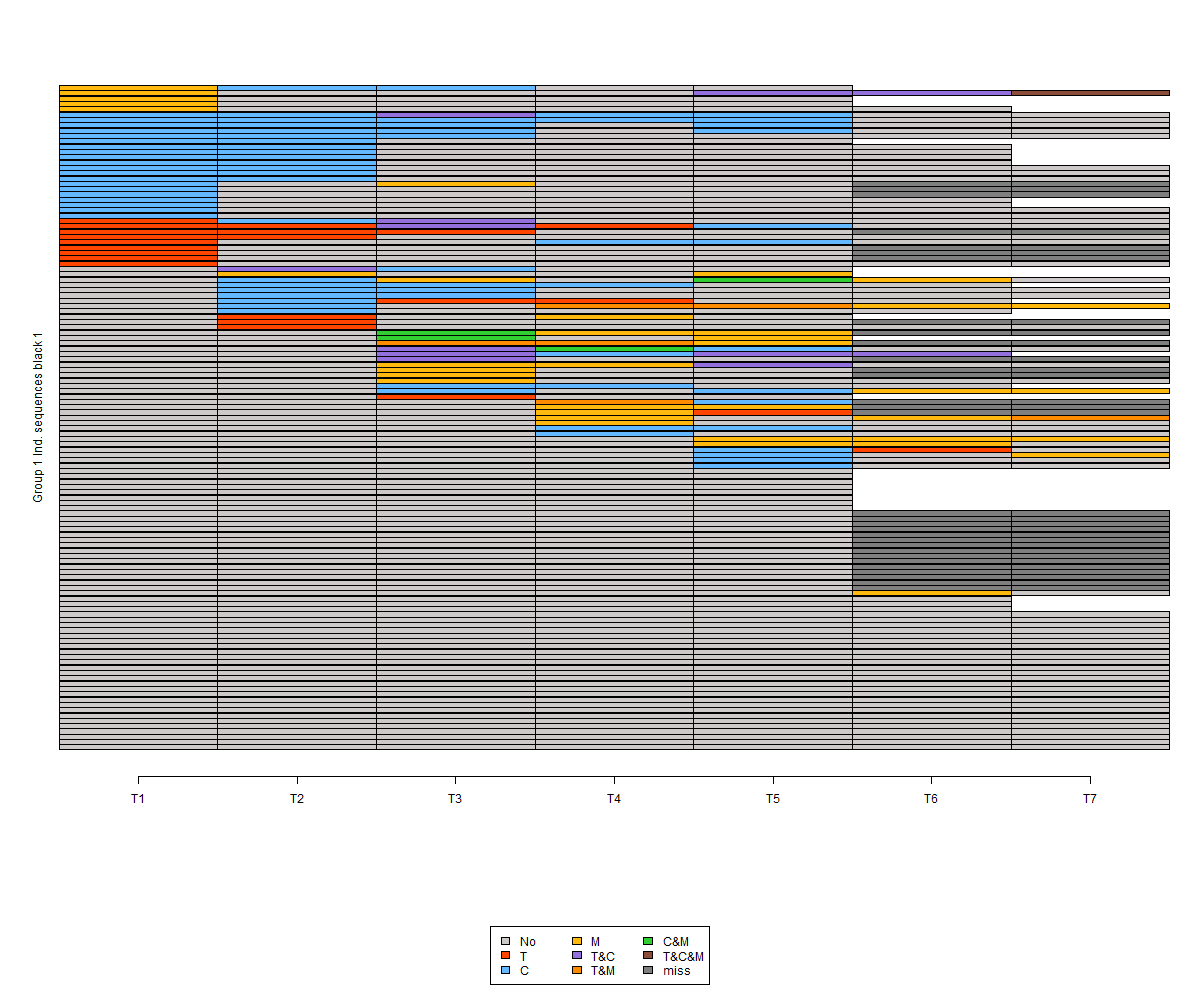


Abbreviations. T = therapy; M = medication; C = counseling

Table S11. The size (N) and the Weighted Average Silhouette Width (ASWw) of each cluster in the five -cluster solution resulting from the sensitivity analysis excluding the COVID-19 years.

| **Cluster** | **N** | **ASWw** | **Description** |
| --- | --- | --- | --- |
| Cluster 1 | 147 | 0.41 | Least intervention |
| Cluster 2 | 66 | 0.50 | Mostly counseling |
| Cluster 3 | 59 | 0.43 | Mostly medication |
| Cluster 4 | 58 | 0.44 | Mixed, mostly C&M |
| Cluster 5 | 115 | 0.10 | Mixed, mostly T |

Table S12. The size (N) and the Weighted Average Silhouette Width (ASWw) of each cluster in the five -cluster solution resulting from the sensitivity analysis in which final missing datapoints were coded as a separate state.

| **5 clusters** | **N** | **ASWw** | **Description** |
| --- | --- | --- | --- |
| Cluster 1 | 143 | 0.48 | Least intervention |
| Cluster 2 | 67 | 0.37 | Mostly counseling |
| Cluster 3 | 79 | 0.36 | Mostly medication |
| Cluster 4 | 82 | 0.40 | Mixed, mostly C&M |
| Cluster 5 | 74 | 0.08 | Mixed, mostly T |

Table S13. Log Likelihood (LL), AIC and BIC values and the size of ethe subgroups for each of the different GBMT models

| **n subgroups** | **LL** | **AIC** | **BIC** | **subgroup size** |
| --- | --- | --- | --- | --- |
| 1 | -5356.24 | 10734,61 | 10829,08 | 445 |
| 2 | 4039.33 | -8023.554 | -7805.107 | (157 ; 288) |
| 3 | 7243.81 | -18709,87 | -18396.96 | (157 ; 194 ; 94) |
| 4 | 15532.49 | -30462,65 | -30037,57 | (104 ; 194 ; 94 ; 53) |
| 5 | 17497.84 | -34869,41 | -34438,42 | (62 ; 95; 68 ; 94 ; 126) |
| 6 | 21708.28 | -37322,3 | -36826,37 | (32 ; 126 ; 86 ; 116 ; 2 ; 53) |
| 7 | 21086.22 | -42008,58 | -41453,61 | (62 ; 51 ; 94 ; 57 ; 86 ; 2 ; 93) |

Figure S4. Visualizations of the intervention trajectories of the five GBMT subgroups, reflecting the intervention use at each timepoint (x-axis T1-T7).


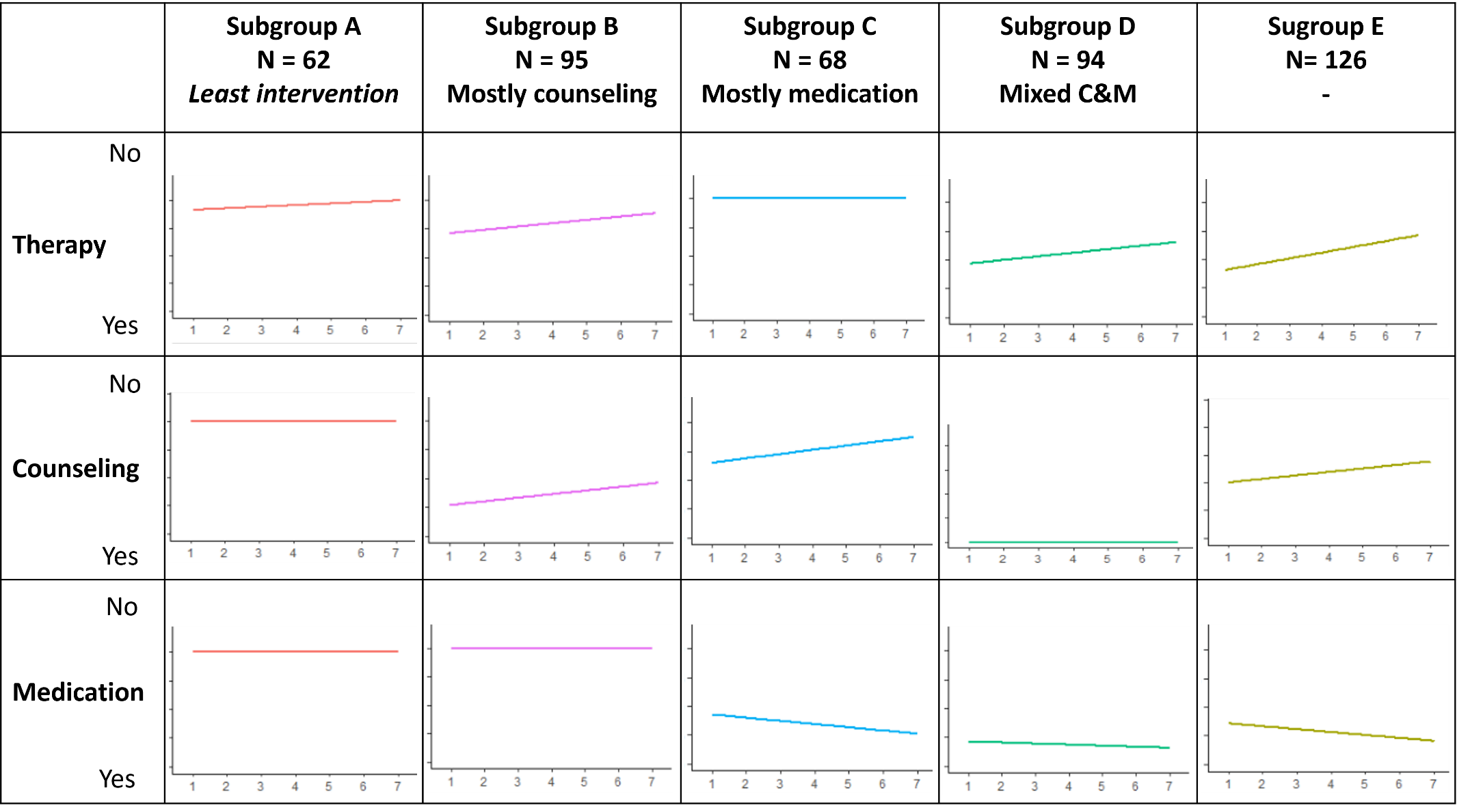


|  | **Least intervention** | | **Mostly counseling** | | **Mostly medication** | | **Mixed,**  **most C&M** | | **-** | **-** |
| --- | --- | --- | --- | --- | --- | --- | --- | --- | --- | --- |
| **Characteristic** | **Cluster 1**  **N = 125** | **Group A**  **N = 62** | **Cluster 2 N = 51** | **Group B**  **N = 95** | **Cluster 3**  **N = 100** | **Group C**  **N = 68** | **Cluster 4 N=84** | **Group D**  **N = 94** | **Cluster5**  **N = 85** | **Group E**  **N = 126** |
| Chronological age |  |  |  |  |  |  |  |  |  |  |
| Mean Age | 52.9 | 53.52 | 47.3 | 47.78 | 49.7 | 48.82 | 47.8 | 46.24 | 44.5 | 49.68 |
| (SD) | (14.7) | (13.91) | (14.4) | (14.75) | (12.4) | (12.78) | (11.3) | (11.97) | (13.0) | (13.41) |
| Age of diagnosis |  |  |  |  |  |  |  |  |  |  |
| Mean Age | 40.5 | 42.02 | 33.3 | 34.64 | 37.7 | 35.60 | 35.2 | 33.74 | 33.7 | 38.91 |
| (SD) | (16.2) | (15.85) | (15.9) | (15.88) | (14.5) | (14.69) | (13.8) | (14.43) | (14.7) | (14.68) |
| AQ score |  |  |  |  |  |  |  |  |  |  |
| Mean | 82.0 | 82.56 | 82.3 | 81.17 | 84.4 | 84.32 | 84.7 | 85.86 | 85.5 | 84.44 |
| (SD) | (11.2) | (12.97) | (9.92) | (9.52) | (10.8) | (10.69) | (10.2) | (10.06) | (10.3) | (10.35) |
| Biological sex |  |  |  |  |  |  |  |  |  |  |
| Female | 36.0% | 30.6% | 62.8% | 56.8% | 60.0% | 58.8% | 65.1% | 69.1% | 71.4% | 57.9% |
| *Educational level* |  |  |  |  |  |  |  |  |  |  |
| Low | 4.35% | 4.26% | 15.4% | 10.9% | 11.8% | 10.9% | 7.46% | 10.4% | 8.96% | 9.57% |
| Medium | 29.4% | 27.7% | 41.0% | 36.1% | 36.8% | 36.4% | 37.3% | 35.1% | 29.9% | 31.9% |
| High | 66.3% | 68.1% | 43.6% | 55.6% | 51.3% | 50.9% | 55.2% | 51.9% | 61.2% | 58.5% |
| Ethnicity |  |  |  |  |  |  |  |  |  |  |
| Dutch | 96.7% | 94.9% | 93.9% | 98.9% | 98.0% | 97.0% | 96.4% | 97.7% | 98.8% | 96.8% |
| With partner | 63.9% | 66.1% | 39.2% | 43.2% | 56.7% | 52.9% | 38.6% | 41.5% | 41.0% | 49,2% |
| Paid employment | 63.8% | 69.4% | 47.9% | 53.7% | 62.8% | 54.4% | 30.4% | 28.7% | 46.9% | 47.6% |
| Co-occurring psychiatric condition | 17.0% | 14.5% | 39.6% | 26.3% | 50.5% | 44.1% | 63.4% | 58.5% | 53.7% | 50.8% |

Table S14. Means, Standard Deviations (SD) and frequencies (%) of descriptive variables for each SA Cluster and corresponding GBMT Subgrou
